# The Evaluation of the Impact of a Stand-Biased Desk on Energy Expenditure and Physical Activity for Elementary School Students

**DOI:** 10.3390/ijerph110909361

**Published:** 2014-09-10

**Authors:** Mark E. Benden, Hongwei Zhao, Christina E. Jeffrey, Monica L. Wendel, Jamilia J. Blake

**Affiliations:** 1 School of Public Health, Texas A&M University Health Science Center, 1266 TAMU, College Station, TX 77843, USA; E-Mails: mbenden@sph.tamhsc.edu (M.E.B.); zhao@sph.tamhsc.edu (H.Z.); mlwendel@tamhsc.edu (M.L.W.); 2 Department of Educational Psychology, Texas A&M University, 4225 TAMU, College Station, TX 77843, USA; E-Mail: jjblake@tamu.edu

**Keywords:** child obesity, school-based intervention, behavior, standing desk

## Abstract

Due to the increasing prevalence of childhood obesity, the association between classroom furniture and energy expenditure as well as physical activity was examined using a standing-desk intervention in three central-Texas elementary schools. Of the 480 students in the 24 classrooms randomly assigned to either a seated or stand-biased desk equipped classroom, 374 agreed to participate in a week-long data collection during the fall and spring semesters. Each participant’s data was collected using Sensewear^®^ armbands and was comprised of measures of energy expenditure (EE) and step count. A hierarchical linear mixed effects model showed that children in seated desk classrooms had significantly lower (EE) and fewer steps during the standardized lecture time than children in stand-biased classrooms after adjusting for grade, race, and gender. The use of a standing desk showed a significant higher mean energy expenditure by 0.16 kcal/min (*p* < 0.0001) in the fall semester, and a higher EE by 0.08 kcal/min (*p* = 0.0092) in the spring semester.

## 1. Introduction

The prevalence of childhood obesity has spread at an alarming rate over the past several decades and currently serves as a significant threat to healthy physiological, behavioral, and psychological child development. According to the Centers for Disease Control and Prevention, obesity affects 17% of the child and adolescent population [1,2,3,4]. Obesity in childhood potentially holds serious consequences for physical health and functioning in later life, as obese children are also likely to be overweight or obese as adults [3]. Due to these major health concerns, targeted prevention and management for the childhood obesity pandemic has become a prominent focus in public health research and practice. 

### 1.1. Complications Related to Childhood Obesity

Children who are overweight or obese are at higher risk for numerous health and emotional problems, including asthma, joint problems, sleep apnea, type II diabetes, high blood pressure, depression, anxiety, low self-esteem, and social problems [5,6,7]. Long-term physiological complications may also include musculoskeletal strain, discomfort, and illness associated with hip, knee, and foot structures [8]. These health problems related to childhood obesity have also contributed to significant increases in healthcare cost in adults, with obesity-related medical treatment amounting to roughly $147 billion in 2009 alone [1]. Academically, children who are overweight or obese are also more likely to repeat a grade in school, exhibit higher absenteeism, experience peer victimization, be placed in remedial classes, and show abnormal scores on behavior rating scales [1,5,7,9]. These behavioral and academic consequences may play a role in later occupational and emotional success. 

### 1.2. School Environment as an Intervention Site

Childhood obesity is generally considered to be influenced by multiple factors, including genetics, family lifestyle, surrounding environment, and parental modeling [10,11]. As a result, applying a single intervention to treat all factors related to childhood obesity poses a significant challenge, as resources are not equally effective or available for all populations. Research particularly shows a higher prevalence of childhood obesity in low socioeconomic (SES) and ethnic minority populations, which may result from limited health education exposure and affordability of certain obesity-targeted interventions [4,12,13]. As a result, treatment has expanded to school environments in which pervasive, sustainable, cost-efficient lifestyle choices can be exposed to children of all weight groups and across all socioeconomic and ethnic enclaves [14].

In 1987, roughly 72% of a sample of elementary school principals reported that they did not believe that school-based interventions were ideal in the treatment of childhood obesity, with some principals expressing doubt that teachers and parents would support such programs [3,15]. Ten years later, a similar sample of principals still identified major barriers in the employment of child obesity prevention programs, including a lack of training, limited materials, potential disruption of classroom time, a lack of funding, and interference with staff time [3]. While the view of schools as an intervention site has drastically changed in recent years, many such barriers still exist and oftentimes lead to timely, costly, or insufficient health programs. 

Modifying school food choices by providing healthier alternatives and limited access to high caloric food is a popular school-based obesity intervention, but it has not shown a consistent relation to long-term eating behaviors and is less frequently identified as an ideal obesity intervention [11,12]. Instead, the best preventive strategy for adult inactivity may be through the establishment of physical fitness patterns during childhood [16]. However, in spite of the presence of physical education and recess time during the school day, time spent in a school setting typically shows a negative association with physical activity [11]. Increased standardized testing and lecture time have also contributed to this negative association, as they create diminished opportunities to be active throughout the school day [17,18]. Children do not tend to compensate for these lost opportunities for physical activity, and prolonged sedentary behavior has become associated with decreased energy expenditure, poor school performance, and negative health outcomes [19,20]. This has resulted in a surge in the perception that sedentary behavior is a significant factor in childhood obesity, with 95.3% of a broad sample of school nurses now reporting concern in this area, compared to only 59% in 1987 [3,21].

### 1.3. Creating Activity-Permissive Classrooms with Stand-Biased Desks

As many obesity interventions can require exorbitant time demands from teachers and administration, modification of classroom settings to create an activity-permissive environment, specifically through the introduction of stand-biased desks, represents a potentially fruitful approach to combatting childhood obesity [22]. Stand-biased, or “standing” desks are passive, low-risk classroom interventions that allow students to sit or stand at their desks during lecture time at their discretion. With these standing workstations, children have the opportunity to increase physical activity and caloric expenditure, as well as to relieve stress on spinal structures that may occur with traditional desks [17,22,23].

Our previous pilot studies showed an increase in energy expenditure and the amount of steps taken within children in a single grade level and school who switched from seated to standing desks in five classrooms over the course of one school year [17,23,24]. Considering the increase of physical activity in the school day has been related to the reduction of childhood obesity, increased caloric expenditure, and heightened academic success in children of all weight groups, examination of cost-effective interventions, such as stand-biased desks, is crucial in the war against childhood obesity [5]. The purpose of the current study is to further examine the energy expenditure and level of physical activity impacted by stand-biased desks in a large sample of elementary school children in multiple grades and schools, across an entire school year. 

## 2. Methods 

Following approval by the Institutional Review Board (IRB) Human Subjects Protection Program at Texas A&M University and the College Station Independent School District Research Review Board (CSISD), 24 teachers representing 480 students from across three elementary schools were consented to the study, with 374 of the 480 students ultimately consenting and assenting at the start of the fall semester as well. Due to funding constraints, a three school model was selected by researchers, which were selected randomly by CSISD from 10 potential elementary schools in the district.

### 2.1. Procedures

**Figure 1:**
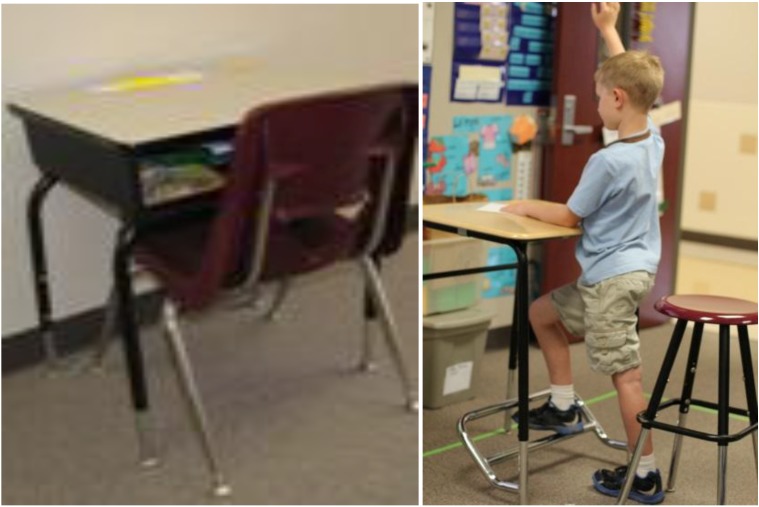
Stand-Biased & seated desk (photos courtesy of PositiveMotion LLC).

A total of eight classrooms went through the consent/assent process to participate in the study from each elementary school. These comprised of two grades, with four classrooms measured per grade. Within each grade at each school, two classrooms were assigned to activity permissive environments, and two were assigned to traditional desks. These traditional desks, Model 2200 FBBK Series by Scholar Craft Products (Birmingham, AL), were provided by the school district, as were the accompanying chairs (9000 Classic Series, by Virco Inc., Torrance, CA, USA. The participants in the treatment condition were each provided with a Stand2learn LLC College Station, TX, USA, stand-biased desk and stool (models S2LK04 and S2LS04, respectively). Figure 1 shows a stand-biased desk and a seated desk used in this study. The full experimental design can be seen in Table 1. Two classrooms in the third grade at School 3, which were originally assigned to the traditional desk group, adopted an alternative seating arrangement (exercise balls for chairs) that did not serve as an appropriate “business-as-usual” control, and were not considered in our final analysis.

**Table 1:** Final number of participating classrooms by grade per school.

Condition	School 1		School 2		School 3 ^a^	
	2nd Grade	3rd Grade	2nd Grade	3rd Grade	3rd Grade	4th Grade
Treatment	2	2	2	2	2	2
Control	2	2	2	2	2 ^b^	2

Notes:^ a^ Fourth grade was measured in lieu of second grade at School 3 in order to better accommodate teachers and administration. ^b^ These rooms were dropped in the final analysis since they adopted an alternative seating arrangement.

Teachers were approached about the study approximately one month prior to the beginning of the school year. During this time, teachers were informed of the study’s purpose, protocol, and financial incentive for their participation. The demands required from the teachers in the study included helping with the distribution of consent forms to their students’ caregivers at the beginning of the fall semester; allowing researchers to take height and weight measurements of participating children once a semester; and to assist with the distribution of armband devices for their participating students for one week in the fall and one week in the spring. All 24 of the teachers who were approached consented to the study.

At the beginning of the fall semester, consent forms were distributed by the participating teachers to each of their student’s caregivers. In addition, researchers attended “back to school” meetings in order to provide further information on the nature of the study to parents. Consent forms for children were gathered and tallied by the third week of school. Researchers then organized short trips to each classroom during the school day in order to gain assent from consented children and, if the child agreed to participate in the study, researchers measured height and weight with calibrated scales and stadiometers and then recorded birthdates, gender and race in order to calibrate individual Sensewear^®^ devices. These devices are designed to systematically record step count and calculate energy expenditure for every minute that the device is worn; energy expenditure is calibrated individually based on gender, age, height, weight, handedness, and whether the subject smokes as variables. The device is attached to a participant’s upper arm via an adjustable Velcro strap and is only activated when its sensors have skin contact. 

Calibrated devices were labeled with an identification number and, due to limited availability of armband devices, were distributed randomly to four classrooms a week until all were sampled (six weeks), with data upload and recalibration occurring each weekend between distribution weeks. Each participant was asked to wear their Sensewear^®^ device for five consecutive school days. Calibrated devices were delivered to classrooms before school on Monday morning and were retrieved from teachers at the end of the school day on Friday of the same week. Teachers were responsible for assisting students with the armband device and were provided instructions on how the child should wear them (*i.e.*, on the upper arm, with sensors touching the skin) and when the devices should be distributed (*i.e.*, as soon as the child arrives for class that day). Participating teachers received $50 per semester at the conclusion of the armband week as the incentive for participation. This protocol was executed in both fall and spring semesters. 

### 2.2. Statistical Analysis

While the Sensewear^®^ devices were worn by participants throughout the entire school day (approximately 8:30 a.m.–2:30 p.m.), final measures were extracted from two-hour block lecture times. This was to ensure that the data would reflect a time when all students were specifically constrained to their individual workstations for lecture style teaching covering math, history and English. Two-hour blocks in the morning, (typically 9:00 a.m.–11:00 a.m.) were identified by the teachers as being consistent in instruction style and topic across all rooms and were matched by control and intervention rooms. Our first attempt at evaluating the effectiveness of the stand biased desks focuses on the most obvious question, which is whether the intervention makes any impact during the traditional lecture time spent with children at their desks. We did not include other periods in this investigation since children do many different recess and PE programs at each school and in each grade, and including them would have confounded our analysis. We do realize that it is also very important to learn the impact of the intervention on the overall EE of children, so in our future studies we will measure children for 24 h a day and report our results accordingly. Additionally, teachers staggered monitor donning times in the morning and our devices needed at least 10 min to acclimate to the room and the wearer. Therefore consistent data could not be counted on prior to 8:30 a.m. The two classrooms that switched to exercise balls were excluded from the analysis. Summary statistics for the baseline measures (taken in the fall) and participant demographics for both the treatment and control groups were first examined. For each of the outcome measures (EE, steps), an average value was obtained over the five day period for each child during the two hour blocks. The means or frequencies were compared between the treatment groups using a two-sample T-test or a chi-square test, as appropriate. Similar participant demographics were also compared between the children who completed the spring assessment and those who did not complete the study or failed to keep their original treatment plan. In addition, means and 95% confidence intervals were plotted for the two outcome variables between fall and the spring semesters for each treatment group.

A hierarchical linear mixed effects model was used to examine the effect of the stand-biased desks on EE and step counts [25]. The advantage of such a model is that it takes into consideration the correlations of the measurements taken from the same person over five days in both semesters, as well as the correlations among children from the same classroom. This model permitted the use of all subjects in the analysis, including the subjects who had only measurements in the fall, as long as the missing data were completely at random. EE and step counts measured in each one minute interval during the two hour blocks on each of the five days served as the dependent variables in the analysis. The fixed covariates included the treatment group, semester (fall *vs.* spring), gender, grade, race, BMI categories, and the interactions between these covariates and the treatment group. The random effects consisted of a random intercept for each child, which is nested in the random effect of classroom. The significance of the interaction terms and the random effects were examined using a likelihood ratio test. Due to the boundary problem for testing a random effect, an ad hoc procedure (*i.e.*, dividing the *p* value by 2) was used to determine whether a random effect should be kept in the model ([26], page 206). A SAS procedure Proc Mixed, version 9.2, was used to perform the analysis. Empirical variance estimators were used to make the results more robust to misspecifications of the correlation structure. 

## 3. Results

Due to deletion of the exercise ball classrooms, attrition, and incomplete physical data sets, 337 of the 374 child participants provided data on EE and steps during the fall semester, and 326 comprised the final sample size at the end of the spring semester. The assignment of the treatment condition was also not equally balanced among schools and grades due to the fact that two planned control classrooms in the third grade at School 3 used exercise balls in place of chairs, and were thus excluded from the control group. This contributed to an imbalance of the amount of participants within each condition, as 202 students were assigned to the treatment group, while 135 were assigned to traditional seated desks (*i.e.*, control group) in the fall. As exhibited in Table 2, no difference was seen in other demographics across the two conditions, including age, gender, race, and BMI measures. 

The sample was almost equally comprised of males and females, with a mean age of 8.5 years. The majority of participating students were White (approximately 70% in the treatment group and 67% in the control group), while roughly 12% (13%) of the treatment (control) groups were Black. About 11% (10%) of the treatment (control) groups were Hispanic, although participants of Asian American descent were represented more in the control group (8%) than in the treatment group (6%). The mean BMI (percentile) was 17.4 (58.1%) for the treatment group and 17.7 (62.7%) for the control group. Based on CDC BMI percentiles of 85%–95% being overweight and greater than 95% being obese, 16% of the treatment group fell into an overweight domain, while 13% was classified as clinically obese. Similarly, 15% of the control group was overweight, while 16% was categorized as clinically obese. Full descriptors of participants in both treatment and controls groups can be seen in Table 2.

**Table 2:** Baseline characteristics for students participating in the study, expressed in means (standard deviation) or percentages.

Descriptives	Treatment (*n* = 202)	Control (*n* = 135)	*p*-Value *
**School**	--	--	0.0459
School 1 (%)	33.17	21.48	--
School 2 (%)	34.16	44.44	--
School 3 (%)	32.67	34.07	--
**Gender**	--	--	0.9469
Female (%)	50.00	49.63	--
Male (%)	50.00	50.37	--
**Age (years)**	8.45 (0.84)	8.49 (0.84)	0.6691
**Grade**	--	--	0.2170
Grade 2 (%)	40.10	40.74	--
Grade 3 (%)	45.05	37.78	--
Grade 4 (%)	14.85	21.48	--
**Race**	--	--	0.4728
Black (%)	11.88	12.59	--
Hispanic (%)	11.39	10.37	--
Asian (%)	6.44	8.15	--
Native American (%)	0.00	1.48	--
White (%)	70.30	67.41	--
**BMI**	17.44 (3.26)	17.73 (3.00)	0.4096
**BMI percentile**	58.11 (30.96)	62.66 (27.95)	0.1704
**BMI Category**	--	--	0.7502
Normal or Under (%)	70.79	68.89	--
Overweight (%)	15.84	14.81	--
Obese (%)	13.37	16.30	--

* *p*-values are obtained using Pearson chi-square tests or two-sample T-tests as appropriate.

We also compared the children who completed the spring assessment with those who dropped out of the study. Included also in the group of non-completers were the two classrooms that used exercise balls for chairs. The results are shown in Table 3. No differences were observed in age, gender, or BMI between the completers and non-completers. However, there were a higher percentage of Black participants in the non-completers group (27%) compared to the completer group (10%).

**Figure 2:**
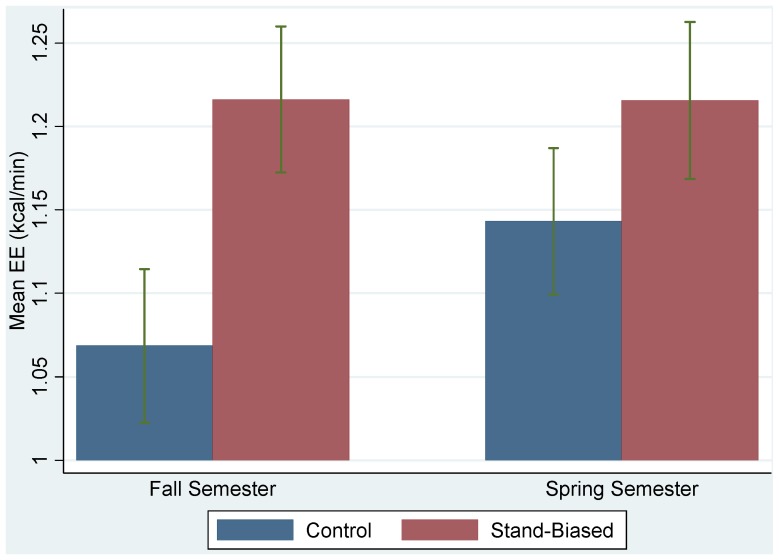
Plots of mean EEs and their 95% confidence intervals for treatment (stand-biased) and control groups in the fall and spring semesters.

**Figure 3:**
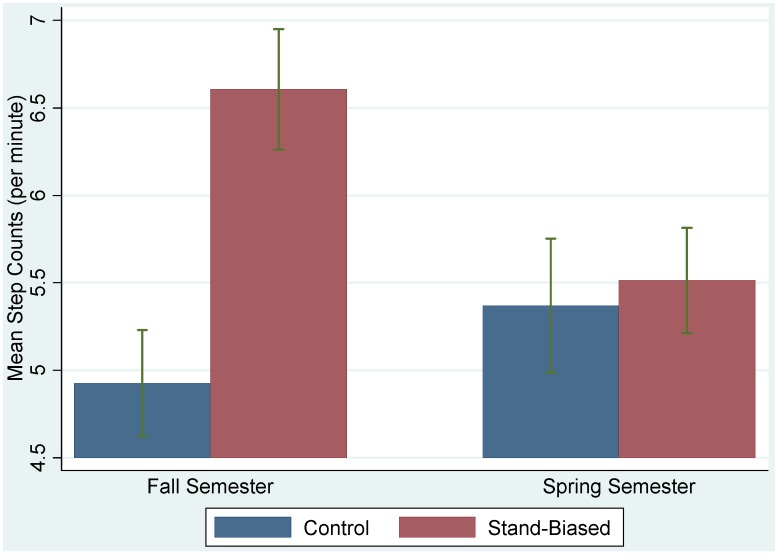
Plots of mean step counts and their 95% confidence intervals for treatment (stand-biased) and control groups in the fall and spring semesters.

The mean and 95% confidence interval plots for the mean EE for different treatment groups in the fall and spring were shown in Figure 2. Similar measures for the step counts were shown in Figure 3. Without adjusting for other covariates, the observed EE and step counts were larger for the treatment group in the fall, but the differences between the two groups were reduced in the spring. 

**Table 3:** Baseline characteristics comparing students who completed the physical measurement in the Fall and Spring (completers) with students who dropped out from the study or changed the treatment (non-completers), expressed in means (standard deviation) or percentages.

Descriptives	Non-Completers (*n* = 48)	Completers (*n* = 326)	*p*-Value ^a^
**School**	--	--	<0.0001
School 1 (%)	68.75	28.22	--
School 2 (%)	6.25	38.96	--
School 3 (%)	25.00	32.82	--
**Gender**	--	--	0.3334
Female (%)	43.75	51.23	--
Male (%)	56.25	48.77	--
**Age (years)**	8.50 (0.67)	8.45 (0.83)	0.7008
**Grade**	--	--	0.0009
Grade 2 (%)	20.83	41.10	--
Grade 3 (%)	70.83	42.02	--
Grade 4 (%)	8.33	16.87	--
**Race**	--	--	0.0400^b^
Black (%)	27.08	10.12	--
Hispanic (%)	8.33	10.74	--
Asian (%)	4.17	7.98	--
Native American (%)	0.00	0.61	--
White (%)	60.42	70.55	--
**BMI**	18.00 (2.77)	17.56 (3.22)	0.3655
**BMI percentile**	67.49 (28.22)	59.55 (30.01)	0.0854
**BMI Category**	--	--	0.3707
Normal or Under (%)	62.50	70.25	--
Overweight (%)	22.92	15.03	--
Obese (%)	14.58	14.72	--

Notes:^ a^ *p*-values are obtained using Pearson chi-square tests or two-sample T-tests as appropriate. ^b^ *p*-value is obtained using Fisher exact test due to small expected numbers in some categories.

The results from fitting a hierarchical linear mixed effects model for EE are shown in Table 4. All the interaction terms between treatment group and other covariates were not statistically significant except the interaction term of treatment and semester from a likelihood ratio test. Both the random effect for child and the random effect for classroom were significant and kept in the model. Therefore, after adjusting for other covariates, the use of a stand-biased desk showed a higher mean EE by 0.16 kcal/min (*p* < 0.0001) in the fall semester compared to the control group, but a smaller difference of 0.08 (*p* = 0.0092) in the spring semester. There was a small, but statistically significant, increase of EE of 0.07 kcal/min (*p* = 0.0157) from the fall to spring semesters, for the control group, which may reflect the natural EE increase that children adopt across their growth cycle. Male students possessed a higher, but not statistically significant EE, when compared to female students (0.04 kcal/min, *p* = 0.0704). Second graders had lower EE than third graders (0.13 kcal/min, *p* < 0.0001), while fourth graders had greater EE than third graders (0.12 kcal/min, *p* = 0.0016). Among the different ethnicity groups, Black participants had a higher EE than White participants (0.06 kcal/min, *p* = 0.0131). It is noteworthy that, compared to participants within a normal weight range, overweight participants showed higher EE of 0.24 kcal/min (*p* < 0.0001), while the EE of obese participants were higher by 0.40 kcal/min (*p* < 0.0001). This reinforced the observation that overweight and obese participants expended greater energy than normal weight participants, regardless of treatment options, or time of the measurement.

**Table 4:** Effects of treatment and other covariates on EE from fitting a linear mixed effects model to the data.

Covariates	Parameter Estimate	Standard Error	*p*-Value
Fixed Effects			
Intercept	0.98	0.04	<0.0001
Treat	0.16	0.04	<0.0001
Semester	0.07	0.03	0.0157
Male	0.04	0.02	0.0704
Grade 2	−0.13	0.03	<0.0001
Grade 4	0.12	0.04	0.0016
Black	0.06	0.02	0.0131
Hispanic	−0.02	0.04	0.5975
Asian	−0.04	0.05	0.3859
Overweight	0.24	0.03	<0.0001
Obese	0.40	0.05	<0.0001
Treat*Semester	−0.08	0.04	0.0459
Random Effects			
Classroom	0.002	0.001	--
Child	0.041	0.003	--
Residual	0.250	0.0006	--

The results from fitting a hierarchical linear mixed effects model for steps were shown in Table 5. Again, all the interaction terms between treatment group and other covariates were not statistically significant except the interaction term of treatment and semester from a likelihood ratio test. Both the random effect for child and the random effect for classroom were significant and kept in the model. We concluded that, after adjusting for other covariates, the use of a stand-biased desk showed a higher mean step count by 1.61 steps/min (*p* = 0.0002) in the fall semester compared to the control group, but the difference diminished to 0.12 steps/min (*p* = 0.8193) in the spring semester. There was a slight, not statistically significant increase of 0.40 steps/min (*p* = 0.4610) from the fall to spring semesters, for the control group. Male students showed a higher step count when compared to female students (0.61 steps/min, *p* = 0.0059), while grade level did not appear to make a difference in step counts. Among the different ethnicity groups, Black and Hispanic participants had a higher step count than White participants (1.36 steps/min, *p* = 0.0001, and 0.62 steps/min, *p* = 0.0041, respectively). Similar to EE, when compared to participants within a normal weight range, overweight participants had a higher step count of 0.78 steps/min (*p* < 0.0001), while the difference between obese participants and normal participants was 0.62 steps/min (*p* = 0.0059). This result showed that overweight and obese children took more steps than normal weight children under both treatment conditions and during each of the semesters.

## 4. Discussion

To the authors’ knowledge, this study is the first in the United States to examine a large-scale, multi-school, multi-grade level cohort of elementary school children and their participation in a stand-biased desk intervention to increase physical activity. Previous pilot studies and this current research indicate that, when children are given environments that encourage and promote movement during instructional time, they will move more and consequently increase their energy expenditure. Similarly, traditional seated environments may actually constrain children’s natural physicality and restrict their movements to a minimal level. This can result in self-perpetuating sedentary behavior that is difficult to counteract in later life. It is important to note that obese children in standing-desk classrooms are not necessarily moving at a rate that is greater than their normal-weight peers, but instead are increasing their activity to be at a similar level. This increased activity, in combination with greater body mass, results in higher energy expenditure that would not have likely occurred in traditional seated environments. In addition, there was no evidence gender or racial variations in the effectiveness of this intervention, although some baseline differences with these factors and physical activity at certain ages, along with the outcome of obesity, should be noted. Overall, the likelihood of this intervention to improve physical activity seems to be equivalent across gender and racial groups without interfering with the students’ comfort, classroom engagement, and behavior [27]. As a result, stand-biased desks are a potential intervention for increasing energy expenditure, especially given the aforementioned difficulty of finding such interventions that uniformly benefit children of all communities and ethnic backgrounds [13]. 

**Table 5:** Effects of treatment and other covariates on step counts from fitting a linear mixed effects model to the data.

Covariates	Parameter Estimate	Standard Error	*p*-Value
Fixed Effects			
Intercept	4.07	0.34	<0.0001
Treat	1.61	0.43	0.0002
Semester	0.40	0.54	0.4610
Male	0.61	0.22	0.0059
Grade 2	0.48	0.41	0.2378
Grade 4	−0.13	0.35	0.7101
Black	1.36	0.36	0.0001
Hispanic	0.62	0.22	0.0041
Asian	0.11	0.26	0.6791
Overweight	0.78	0.19	<0.0001
Obese	0.62	0.22	0.0059
Treat*Semester	−1.49	0.66	0.0246
Random Effects			
Classroom	0.46	0.19	--
Child	2.56	0.22	--
Residual	164.01	0.38	--

The concept underlying the study design was to show how the environmental change of a stand-biased classroom might “appear” at the start of a new school year. In this situation, teachers may not have the opportunity to receive formal orientation and training to the desks. This was designed so the researchers would maintain the lowest level of interaction possible with the participants to determine if there was a measurable treatment effect. As a result, teachers were purposefully provided limited information and training with the desks in order to avoid this potential bias in data collection. 

This study was conducted in elementary school children, whose school schedules were comprised of a range of diverse activities, including relatively short periods of focused instructional time interchanged with art, music, computer, physical education, lunch, and recess classes. As children progress to higher grade levels, however, seated instructional time typically increases, with up to six hours a day usually spent sitting in didactic-format classrooms. The effects of stand-biased desks may, therefore, be greatly magnified with older students whose school days are characterized by even more sedentary activities. Further study is needed to explore this hypothesis.

These findings also have obvious implications for policy and practice. The results of this study and previous pilot studies have established that activity-permissive classrooms do not cause harm to students; result in increased energy expenditure that may combat obesity among those in the highest risk categories; and improve behavioral engagement [17]. If this environmental change improves both health and academic outcomes, this should serve as an incentive for schools to invest in altering their standard for classroom furniture to stand-biased modifications. 

While the findings of this study are significant, several limitations should be considered. First, the study was conducted only in elementary school students, and the results are not necessarily generalizable to populations in different developmental stages (*i.e.*, adolescents). Second, while the schools participating in the study were diverse, the demographics of the students participating and the school settings may not be representative of those in other communities. Third, a lack of baseline measures for all children, in all schools, with all teachers from our cohort, is a limitation of this study. More research is needed to understand how different demographic and school characteristics would influence the overall effectiveness of activity-permissive classrooms. Finally, the analysis was limited to the two hours each day that were comparable for all the classrooms during instructional time. Additional data would be needed to monitor effects throughout the day. Future research should include larger scale studies, 24/7 monitoring and older students. Despite the limitations, this study offers important evidence that stand-biased environments can contribute to increasing EE for school children.

## 5. Conclusions 

In conclusion, the findings of this current study indicate that stand-biased desks implementation had a statistically significant and positive impact on EE and steps for children during the standardized lecture time. The downward change of step count in the spring semester in the treatment group may have been related to acclimatization (aka familiarization) to the intervention over time. If this is, in fact, a case of reversion to old habits after the new intervention has worn off, it may indicate a need for more overt training and education on the use and purpose of the standing feature of the desks. Further studies are needed to establish this time related effect and the impact that training and education might have on physical behavior and thereby EE. 

Affecting the energy balance is critical to changing the trajectory of obesity. In light of the *Healthy People 2020* goal of reducing childhood obesity, Wang, Orleans, and Gortmaker [28] suggest that a 41 kcal/day increase in EE would be sufficient for changing the obesity trend among the child population. This intervention suggests that a 41 kcal/day increase is not out of reach for this population due to a simple environmental change in a school setting, where most young people spend the majority of their day (a mean of 0.16 kcal/min difference (treatment *vs.* control) is equal to 19.2 kcal/2-hour study period). Ultimately, stand-biased desks as an activity-permissive environment, show promise as an EE intervention for children in school. 
